# Sensory, Affective, and Social Experiences with Haptic Devices in Intramural Care Practice

**DOI:** 10.3390/nursrep14010019

**Published:** 2024-01-23

**Authors:** Dianne Vasseur, Sima Ipakchian Askari, Sandra Suijkerbuijk, Henk Herman Nap, Wijnand IJsselsteijn

**Affiliations:** 1 Vilans, Centre of Expertise Long-Term Care, P.O. Box 8228, 3503 RE Utrecht, The Netherlandsh.nap@vilans.nl (H.H.N.); 2 Department of Industrial Engineering and Innovation Sciences, Eindhoven University of Technology, P.O. Box 513, 5600 MB Eindhoven, The Netherlands; s.suijkerbuijk@tue.nl (S.S.); w.a.ijsselsteijn@tue.nl (W.I.)

**Keywords:** tactile, COVID-19, haptic devices, telecommunication, intramural care facilities, profound intellectual and multiple disabilities, dementia

## Abstract

Previous work has shown that technology can facilitate the communication of emotions, social touch, and social presence through haptic devices, meaning devices that provide a haptic stimulation. However, for special user groups living in long-term care facilities, such as people with dementia, the applications of these haptic devices are not apparent. The aim of this study is to understand how haptic devices can be used in intramural care facilities in times of social isolation, focusing on haptic devices that utilize haptic sensation. Five haptic devices were tested at three care facilities. Insights from this study highlight the potential of haptic devices to enhance sensory, affective, and social experiences during video calling between residents and their relatives. Moreover, the importance of the tactile sensation and form factor of haptic devices in the care context is addressed, along with insights on how to create the appropriate atmosphere during video calling.

## 1. Introduction

Direct interpersonal contact through social touch is important for human beings. It plays an important role for human development, interpersonal communication, social communication, and social attachment [1,2,3]. Social restrictions due to the COVID-19 pandemic, such as social distancing, curfews, and quarantines, impacted this human need on a global scale. These restrictions had a major negative influence on the wellbeing of people in intramural care facilities, such as people with dementia or people with profound intellectual and multiple disabilities (PIMD). Residents were, for example, prevented from socially engaging with their loved ones [4]. The absence of these visits was associated with an increase in feelings of loneliness and a lower overall quality of life [5]. Care facilities and family members have expressed their concerns regarding premature deaths potentially caused by the lack of social contact during family visits [6,7].

During the COVID-19 pandemic, creative solutions were developed to facilitate touch interactions for the populations in residential care, for example, by means of ”cuddle screens”. These allow the hugging of a loved one through a physical barrier designed to prevent infections [8]. When intramural care residents were unable to engage in direct interpersonal contact, telecommunication was regarded to be beneficial as well [4,9]. Telecommunication, such as video calling, helped with maintaining social networks, receiving stimulation, and for reaching help when needed [10,11,12].

Despite these benefits, existing telecommunication solutions have not been meeting all the needs of residents in intramural care facilities and of their family members. Communicating through a device can be challenging for this group of people. People with dementia, for example, can experience video calling as overwhelming, confusing, or too complex [9]. In addition, telecommunication is unable to fully compensate for direct interpersonal contact [4,5,13,14]. The remote interactions through telecommunication merely convey auditory and visual cues, while tactile cues of interpersonal contact are missing. Creating a ”feeling of presence” is often regarded as the stumbling block for regular telecommunication solutions, such as a telephone or videoconferencing [15], due to the absence of touch and the inability to make real eye contact [16]. It is likely that the absence of touch makes it harder to pursue actual interpersonal contact in remote conversations, given the substantial role of the senses in social interactions. This may probably be even more important for populations who experience difficulties in verbalization or have cognitive problems [17,18]. Moreover, it has been shown that communication through a tactile modality can lead to more personal conversations [19].

Various technological solutions have been developed that facilitate the communication of emotions, social touch interactions, and social presence through haptic or tactile interfaces (i.e., warmth, pressure and/or vibrations) that operate over a distance, the so-called Affective Haptic Devices or AHDs [19]. In addition to AHDs (which utilize haptic and tactile displays to facilitate communication between persons), there are also haptic technologies that provide a sensory stimulation to a person without such a communication element. In this paper, both forms of haptic technology will be referred to as haptic devices. Although the effectiveness of these devices for the general population has been shown [19,20], less is known about whether these haptic devices can enhance the experience of video calling between intramural care residents and their family members in daily practice. This would be valuable to investigate, as the inclusion of haptic devices could create a more personal and richer experience of video calling. Therefore, in the current study, we explored five different haptic devices conveying three different types of stimuli: thermal, vibrotactile, and force/pressure. In this paper, we decided to focus on haptic devices that utilize the haptic sensation due to the importance of this haptic modality for interpersonal communication [1]. 

The aim of this study is to further understand how haptic devices can be used during video calls to enrich remote communication between residents in intramural care and their relatives in times of social isolation. For this research question, we explore which product attributes are perceived as valuable for remote communication in order to contribute to the existing Human Computer Interaction (HCI) research in this field. Furthermore, the current research illustrates how people living in Dutch care facilities and their relatives can experience enriched video calls focusing on three different levels of experiences, which are based on existing interaction models from the HCI literature [21,22,23].

(a) Sensory experience—how is it perceived?
(b) Cognitive affective experience—how does it make them feel?
(c) Social experience—how are they linked to another social actor?

In addition, we aim to provide new insights into the possible obstacles and challenges that care professionals face when making use of haptic devices in their daily practice. Their experiences with such devices were studied in the first months of the year 2021, a period when care facilities were still limiting access. We adopted the research methods accordingly by using on- and offline focus group sessions, guided observations and interviews by care professionals, and additional on- and offline interviews with family members and care professionals.

## 2. Related Work

There is a wide range of interventions that involve the sense of touch in intramural care. For example, touch therapy [24], the use of animal-like robots [25,26,27], or through devices that provide a multisensory stimulation (e.g., [28,29,30]. An example of the latter is “snoezelen”, which refers to a multisensory environment where people can immerse themselves in various pleasurable sensory stimulations. These devices have been shown to be effective in evoking positive emotional responses from its users (e.g., [18,31,32,33]) and, in some cases, even enhancing social interactions [33]. 

Such sensory interventions and devices provide a haptic stimulation. In order to understand how such haptic stimulations can impact the experience of a video call, it is important to understand the different levels of experiences that exist. A user, for example, can have a rather passive interaction with the device. In this case, they likely experience merely the sensation provided, but they are not under the impression that they have had a social interaction with another person (e.g., in the case of “snoezelen”). Within the field of HCI, various models have been suggested regarding these levels of interaction experiences [21,22,23]. We considered which levels might be applicable and valuable for residents in intramural care facilities (e.g., people with dementia or profound intellectual and multiple disabilities (PIMD)). In this paper, we therefore identified three levels of experience: sensory, affective, and social experience, which will be further discussed in this section.

### 2.1. Sensory Level

The sensory level encompasses the tactile sensation, meaning how the sensation feels (e.g., temperature and texture), as well as the form of the device, encompassing the physical shape and materials used [34]. In the field of haptics, various prototypes have been developed using different actuation types [35,36,37]. For example, the TaSST [38] is a wearable sleeve that uses vibrotactile stimulation to provide users with the sensation of a stroke. An example of a device using warmth is the ThermoCaress [37], which uses a thermal stimulus to strengthen a stroking sensation on the user’s forearm. In addition, there are haptic devices that use an actuation-type force; for example, the Hey Bracelet [36], which can be used to send a “squeeze” to a loved one. Regarding the implementation of haptic stimulation for people with dementia, previous work has shown the use of interventions such as deep-pressure massages, soft stroking movements on the skin, and weight-induced pressure by weighted products [24].

When looking at the form factor of the haptic device in the field of haptics, various prototypes have been developed that incorporate human-like and/or organic shapes. For example, The Hug [34] is a haptic device that facilitates the sending and receiving of haptic messages. The shape of The Hug resembles the form of a human body. This makes it very natural to hold The Hug and very intuitive for users to know how they can interact with the device. Two other examples of haptic devices with an organic shape are the Frebble [39] and the VibroBod [40]. The Frebble is designed in a shape that invokes users with the experience of holding hands. Pilot studies with the Frebble have shown that participants were able to intuitively understand how they should hold the Frebble [39]. The VibroBod [40] is a haptic device that can be placed on one’s lap. The shape of the VibroBod was designed with the purpose of giving users the sense of holding on to someone or being held. In addition to these organically shaped haptic devices, there have also been devices designed that make use of soft materials to create a pleasant tactile experience when touching the haptic device; for example, Paro [41], Huggable [42], and the “Haptic Creature” developed by Flag and MacLean [43].

### 2.2. Affective Level

The sensory level can have an influence on the affective level of a haptic device, meaning that the sensory properties of the haptic device can influence the affective state of a user. Past research on haptic communication has focused mainly on the bottom-up responses to receiving a touch [44,45]. These bottom-up responses involve the perceptual qualities of a touch; for example, the speed of a touch [45]. A touch with a speed between 3–5 cm/s is typically perceived as more pleasant compared with a touch at a lower or higher speed [46]. Moreover, it has been suggested that the use of force is a more appropriate actuation type compared with vibration for haptic devices used for affective communication [44].

Along with the perceptual qualities of a haptic sensation, the form factor of a device might also have an effect on a user. Past designs have been shown to induce aversive reactions due to their form factor. An example of such a design is the MobiLimb [47], which was perceived as uncomfortable by some people due to its realistic and human-like design. Moreover, it has been shown that, for care technologies, the form factor of a device can play a role in the acceptance of the device [48,49,50]. For example, prominent alarm buttons might be experienced as stigmatizing; as a result, people might be hesitant to wear them [48]. Therefore, alternative form factors have been suggested; for example, implementing alarm buttons and/or trackers in a smartwatch [50] or jewelry [51,52].

Alongside research that focuses on how the sensory properties of a haptic device can influence the affective state, there have also been quite a number of studies investigating the effectiveness of haptic devices in improving the quality of life and emotional wellbeing of their users. Past research suggests that robot pets can have a positive effect on the quality of life and lead to higher feelings of pleasure among users [53]. A study on the “Haptic Creature”, a robot pet aimed at calming people down, was also found to be effective in increasing the levels of relaxation and happiness [54]. Similarly, it has been shown that “snoezelen” can lead to positive affective responses. Research has shown that “snoezelen” can have various positive effects, such as more spontaneous behavior by patients [29,55], and has a positive influence on one’s social and emotional wellbeing. In addition, research showed that providing haptic stimulation to people with moderate to severe dementia can stimulate relaxation [28]. Weighted products, such as vests or blankets, are associated with positive effects on the mental states of people living with autism, anxiety, or dementia [56] and are used in touch therapies [24]. 

### 2.3. Social Level

The last level of interaction focuses on whether the haptic device can enhance the social engagement between people during the mediated interaction. This can be achieved in various ways. One way that a haptic device could enhance social engagement is because a person links the haptic sensation to another “entity”, meaning that the user is under the impression that they are receiving this sensation from another person or avatar/robot rather than only perceiving it as stimuli (as would be the case for the sensory and affective level) and that they are having a social interaction with this other entity, i.e., an experience of a so-called social presence [57]. Such an interaction can refer to both a human-to-human interaction and human-to-machine (e.g., robot pets) interaction. Past research has suggested that people are able to have a social interaction with robot pets. It has been shown that, for example, in the case of Paro—a robot seal—people are able to have a social interaction with Paro [31]. Moreover, the use of Paro was associated with a variety of benefits for the users (e.g., Paro was able to put restless and sad persons at ease; [58]). Although robot pets such as Paro have been shown to be effective in evoking some social experience when interacting with such a haptic device, there are still some limitations. For instance, the use of an animal in the physical design of a haptic device can result in the user’s perception of the robot pet being dependent on their affinity with animals [59]. 

Moreover, a sensory level is also achieved if the haptic device enriches or improves the social engagement of a communication (e.g., by making the conversation more personal or by acting as a conversation piece). Past research has explored to which extent haptic devices can provide an improved experience for their users. Previous field studies have shown [19,60] that AHDs were able to provide added value to the users. Results showed that people appreciated receiving a message through an AHD, making them feel loved or making them aware that someone is thinking of them. Another example of a haptic device facilitating social engagement is called the SAM [61], which consists of two spheres that can be used in the communal space of a care home. Users can touch the spheres (e.g., holding or shaking them), and then SAM provides a stimulus by vibrating, making sounds or changing its colors. The two spheres mimic each other; for example, if one user shakes their SAM the device will vibrate and show a color. In response, the SAM of another user will mirror this behavior and also vibrate and show a similar color. Through this behavior, SAM can spark conversations between users.

These three layers of interaction (sensory, affective, and social) can be seen as stairs of a staircase, with the sensory level as the lowest stair and the highest stair being the social level. If the sensory level is not sufficient (e.g., unpleasant or not noticeable), it will be difficult to reach the next stair, i.e., the affective level. In this paper, we will use this three-layer model of interaction as a framework to analyze our results.

## 3. Materials and Methods

To understand how tactile modality could be used during video calls in intramural care settings, we performed three different qualitative research activities to access various perspectives on the user’s experiences with haptic devices. The research was conducted in early 2021, with the care facilities still affected by most of the regulations implemented due to COVID-19. First, the research started with online and offline group sessions. Participants from these group sessions were invited to carry out “try-out sessions” [62], an in situ research methodology with participant observations. In addition, the research team conducted several semi-structured interviews with care professionals and family members of residents of intramural care facilities to understand their perspective and experiences. The data for this study were obtained by means of a triangulated approach [63].

### 3.1. Participants

Care professionals, residents, and their relatives from three care facilities for people with disabilities or older adults in The Netherlands participated in the study. One care facility delivered care for people with PIMD and two delivered geriatric and psychogeriatric care (nursing homes). 

In total, 14 care professionals participated across the different studies. Seven care professionals who were involved in the try-out sessions agreed to participate in a follow-up interview as well. Two interview sessions were held in groups, consisting of four and two care professionals, and one interview was conducted in a one-on-one setting. All of them had at least one year of experience working at the specific care organization, amounting to over 13 years of experience. 

**Figure 1:**
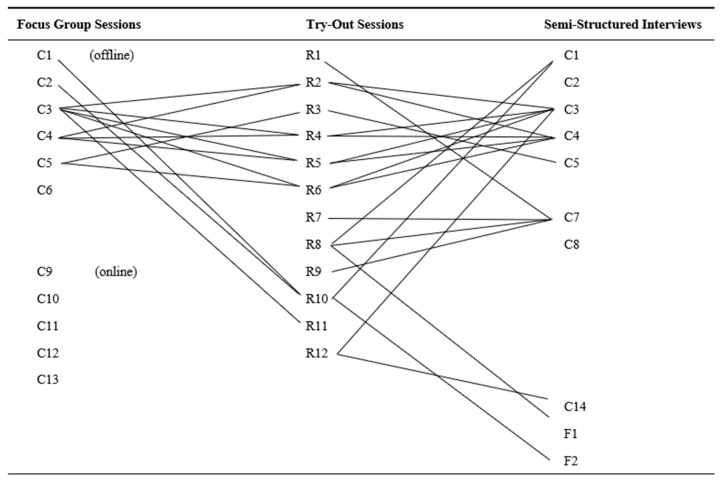
Care professionals (C), residents living in the care facility (R), and family members (F) across the different studies.

Twelve residents were recruited by the care professionals of the participating care facilities. These residents participated in the try-out sessions. Three of the residents lived in a care facility for people with PIMD, and nine participants were residents in care facilities for older adults. The participant characteristics are listed in Table 1. Two family members volunteered to participate in the semi-structured interviews. Both family members were children of people diagnosed with dementia who participated during the try-out sessions. An overview of the participants and their connections can be found in Figure 1.

### 3.2. Materials

The haptic devices were selected in such a way that they covered the three most common actuation types for AHDs, namely thermal, vibrotactile, and pressure stimuli. All materials were commercially available during the study period. These materials were selected as they were considered the most suitable and available materials on the market at the time of the study. A selection was made based on the type of haptic feedback, the availability, and the extent to which the materials could be used during video calls. It was not a requirement for the materials to be specifically designed for usage while video calling, as there were very few options that did not meet the availability requirement. A number of devices were excluded from the initial list, such as the Frebble and the TaSST sleeve, because they were not available on a large scale. An overview of the selected materials is presented in Table 2.

### 3.3. Procedure 

**Figure 7:**
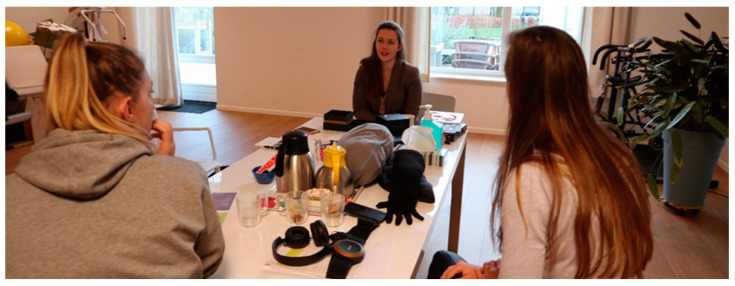
Impression of the physical focus group setting.

First, three focus-group sessions were held, of which one was online and two were offline at the participating care facility. An impression of the offline focus-group session is displayed in Figure 7. The focus group sessions with the care staff started with an introduction to the research, followed by an introduction to interpersonal contact. The discussion particularly focused on what interpersonal contact entails within the context of intramural care and how it has been pursued during the COVID-19 restrictions. Then, the products were presented one by one, functioning as probes to initiate the discussion on haptics in telecommunication and providing the care professionals with some first-hand experience with the products. Participants of the online sessions were limited to images and live demonstrations by the researcher. During the demonstration of the products, participants were asked about their expectations on using the products to foster interpersonal contact between residents and family members. 

As the research took place when COVID-19 regulations were still in force, researchers were not allowed into the care facilities. Therefore, the care professionals who participated in the group sessions were invited to be “researchers” during the “try-out sessions” [62]. For the try-out sessions, a box containing the haptic devices was delivered to the participating care facilities, which included instructions for the study and for using the devices. A checklist was added to the box to help care professionals to keep track of all the required steps during the research activities. 

The try-out sessions were held in one-on-one settings, with only the resident and care professional present. The setting was a calm room where the residents felt comfortable. The products were displayed within the reach of the resident. Then, the care professional was instructed to observe the residents’ initial responses to the different haptic devices. The residents were free to choose which device they wanted to try out first. Also, the order of devices was not fixed, as long as the residents had seen all the devices one by one. Care professionals were instructed to document the observations in a form provided by the researchers. This scheme was based on the validated Video Coding—Incorporating Observed Emotion (VC-IOE) protocol [70]. The VC-IOE protocol was developed to measure the engagement of people living with dementia with a certain stimulus. Six different levels were included, namely emotional, verbal, visual, behavioral, collective engagement, and agitation. The form served as a guide to document the occurrences of certain responses. The form also included space for elaborating on descriptions and duration indications. 

After the first try-out session, the care professionals were instructed to test the haptic devices in combination with a video call with a family member. A selection of two or three devices that were positively received by the residents were introduced during the video call. The family member could make comments or suggestions on the haptic devices being used while video calling. Again, the care professional documented the responses of residents for each of the devices that was tested. Altogether, the sessions took approximately 30 to 60 min each. The care professionals could clarify or elaborate on their documented observations during later interactions with the researchers, in the form of follow-up interview sessions.

**Figure 8:**
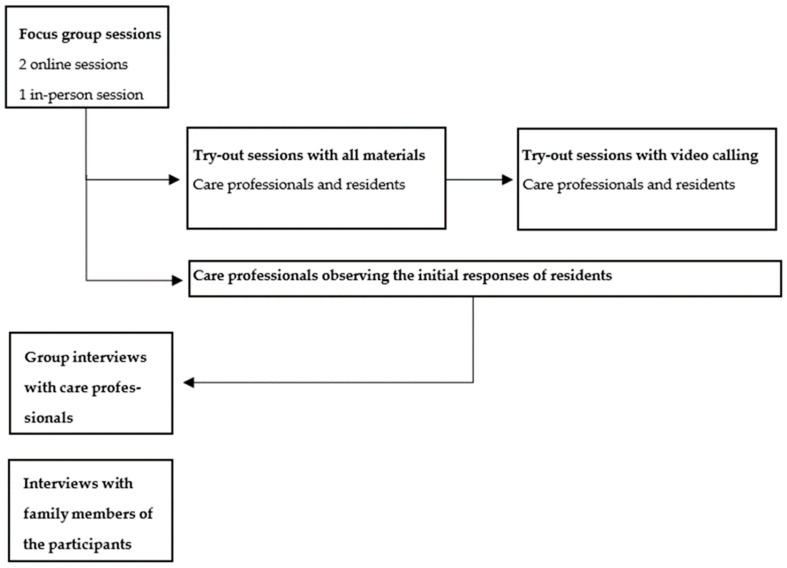
A schematic overview of the procedure.

Four weeks after handing over the box with the haptic devices to the care facility, semi-structured interviews were conducted in small-group settings. Group interviews were held with multiple care professionals from the same care facility. One interview took place in a one-on-one setting, three were held online, and one interview was conducted over the phone. Interviewing the observers and family members allowed for a more comprehensive understanding of the experiences with the haptic devices. The questions asked during the interviews can be found in Appendix A. After the interviews, all participants were thanked for their time and help. A schematic overview of the procedure can be found in Figure 8. All sessions and interviews were originally conducted in Dutch to ensure a comprehensive interaction with the participants.

### 3.4. Data Analysis

The transcripts and field notes of the observations were analyzed by a thematic analysis according to the principles of Braun and Clarke [71,72]. The data were analyzed in Dutch, using the MAXQDA 2020 software, version 2020.4.1 for qualitative research. First, the data from the three different facilities were analyzed separately. The goal of this analysis was to become familiar with the data, gaining insights into how to best structure the data, given the various research activities, contexts and research populations of the collected data. Moreover, the familiarization provided insights into the possible occurrence of overlapping themes for the different residents and care facilities. This preliminary coding scheme included five initial themes focusing on the positive, neutral, or negative expectations and experiences with the haptic devices. Eventually the recurring patterns found in the data and the relations between codes were structured among the different levels of experiences: sensory experience, cognitive affective experience, and social experience. After completing the analysis, all schemes, themes, and quotes underwent translation into English. The translation process occurred post-analysis in order to maintain fidelity to the data. 

Throughout the whole data analysis process, two other researchers were involved in providing feedback on the analysis (e.g., the preliminary scheme and the final themes and subthemes). This included one researcher who was familiar with the data and who was also present during the data collection, whereas the other researcher was only familiar with the research aims. Analyzing the data with input from these two researchers with different perspectives minimized the occurrence of biases. 

### 3.5. Ethical Considerations

The study was reviewed and approved by the Ethics Committee of Eindhoven University of Technology (ERB2021IEIS5 on 19 February 2021). To guarantee the safety and wellbeing of all participants, with special attention to the residents of the care facilities, we considered a careful procedure. First, the recruiting process of residents was led by care professionals. Given their daily engagement with the residents, they were able to assess who would be willing to participate in the study. Family members were introduced to the study as well by means of detailed information leaflets. Due to the COVID-19 regulations and to reduce the risk of contamination, the research team did not directly interact with the residents. The healthcare professionals were instructed to guide the “try-out sessions” by selecting haptic devices that fitted the residents’ interests and desires. This personalized approach was selected to avoid any discomfort or agitation for the participants when using the devices. If possible, the residents provided their written informed consent to participate in this study. In cases where participants were not able to provide written consent, their legal representatives signed the consent forms. Care professionals were instructed to discontinue the sessions when verbal or other signs of non-consent or agitation were given by the residents. In case participants were interested in continuing to use or to keep the materials, the researcher shared the information on where to buy the materials with the healthcare professionals. Data gathered for the study were anonymized by allocating a participant number to the participating healthcare professionals, residents, and family members.

## 4. Results

In the following section, we will present the themes, subthemes, and underlying relationships that emerged from the data analyses. These are structured among the three levels of interaction with the haptic devices. First, the sensory level includes themes related to tactile and physical experiences with the haptic devices. The second level pertains to the themes regarding the affective experiences with the haptic devices, followed by the potential social effects of these devices in the third level. Translated quotes from professional caregivers (C1, C2 …, C14), residents (R1, R2 …, R12), and family members (F1, F2) are included in the findings. The original Dutch quotes have been translated to English by the authors. The term “residents” is hereby used to describe both people living in nursing homes as well as for people with PIMD living in group homes. In the event that differences are found between the two groups, the distinction is explicitly mentioned. 

### 4.1. Sensory Level 

On a sensory level, the insights show different elements of the perceived experiences of care staff, residents, and family members in the use of haptic devices. In the following sections, we illustrate how the different haptic stimuli were valued, how natural form factors appeared to be the most preferred by the participants, and the perceived difficulties with the more technological haptic devices.

**Valuing the strong sensations of pressure and warmth stimuli**

*“They occasionally said ‘I liked the warm feeling’, so they remembered something about the products. Not the product itself but its effects”*.

(C14)

Participants in our study reported that they valued the use of physical warmth and pressure in the haptic devices. Firstly, it was found that warmth acted as an attracting force, as participants were captivated by feeling the warm surfaces: *“It seemed like people were naturally drawn to it [the warm hand], that was quite special”* (C14). Moreover, care staff reported during the try-out sessions that some of the residents made sure that the warm surface of the hand or collar were touching their bodies. Secondly, the pleasure of feeling warmth and pressure became evident through the fact that multiple participants reported that these stimuli had a calming effect on participants. From the interviews, care staff reported identifying signs of increased relaxation when residents held warm and weighted products. Sitting or lying down with the weighted collar, for example, could help some of the participants with adopting a more relaxed state of mind. In one instance, a care staff member reported that it also helped with decreasing muscle tensions: *“Her posture became more relaxed. It was very nice to see the effect it [the weighted collar] had on her”* (C3). In addition to observations from the caregivers, some residents also verbally expressed their pleasure with the sense of warmth or pressure, as illustrated by the quote above by C14.

However, the vibrations and pressures were not always perceived well by the participants. One care professional mentioned that she thought that the resident was reluctant to use the device because the sensation of the vibrations was not pleasant and was unfamiliar: *“I’ve tried it with a resident, but she thought it was a bit scary because it uses a small vibration. Most of the residents thought it was less pleasant to wear”* (C14). Moreover, the vibrations and pressure provided through the Hey bracelet and the Hug pillow were reportedly too subtle for the participants. As a result, the products were not able to evoke any responses from participants: *“I am wondering whether the breathing pattern is strong enough for him to sense it”* (C1). Care professionals mentioned that participants would need to completely focus on the haptic sensations in order to feel them. Dividing their attention over the different stimuli could have been too difficult. As a result, some of the more subtle sensations were lost during the conversation: *“When we are in a conversation, you are less able to focus on what the product is doing”* (C1). 

Indeed, when products were used independently of telecommunication, observers mentioned that participants were focused on the haptic sensations. This is illustrated by one participant who used the music strap in a standalone fashion. The vibrations appeared to be strong enough under these circumstances. The observing caregiver noticed positive responses toward feeling the vibrations, especially in terms of engagement and movement: *“It [haptic device] also challenged them to move”* (C2). This suggests that sensations coming from the haptic devices could provide value for people with PIMD. However, this is on the condition that the sensations are either strong enough to notice or are presented apart from other stimuli sources.

**Residents tend to prefer more natural haptic devices**

*“When something looks touchable, it is interesting for people. For example, the hand is clearly something that people like to hold and something that looks inviting to touch. I think there is a large difference compared to the bracelet or strap, which are far less appealing for our people”*.

(C8)

**Figure 9:**
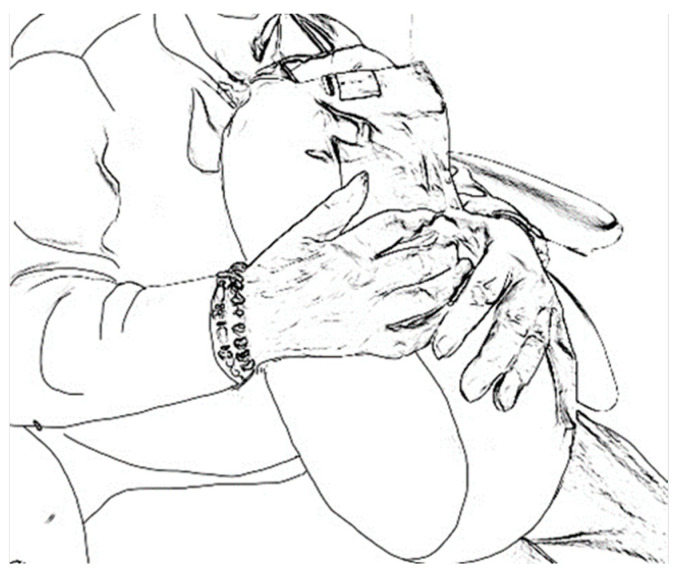
Resident holding the hug robot on her lap.

**Figure 10:**
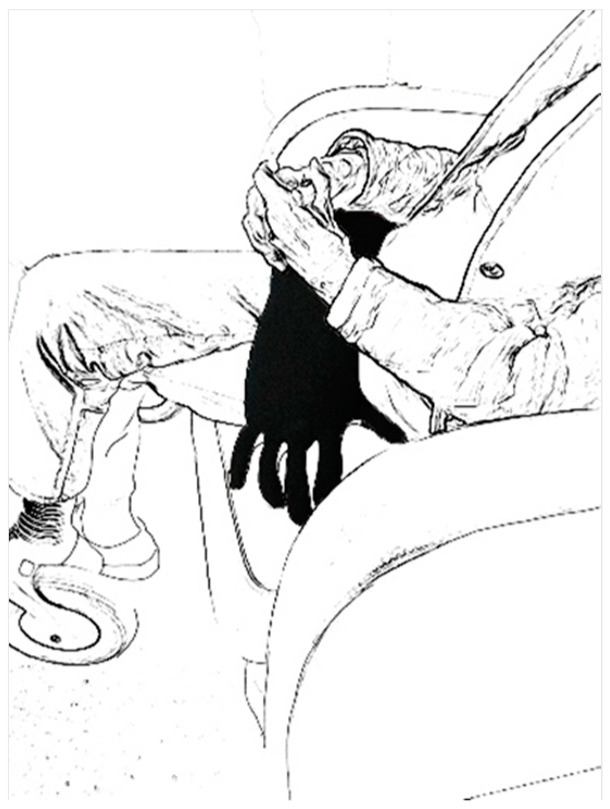
Resident holding the warm hand on her lap.

The observed responses of residents demonstrated a preference for familiar, organic shapes and devices that elicited tactile pleasure. Residents showed, as reported by the caregivers, a higher interest in trying products with a more natural appearance (e.g., the warm hand). This became evident in two ways. First, residents more frequently selected devices with natural shapes: *“The resident was more likely to say things like ‘can I hold this?’, or ‘that looks nice’, so that was a more natural process”* (C14). Second, the preference for natural devices was also prevalent from the residents’ responses during video calls. They intuitively held on to these devices or placed them on their laps during video calls, as shown in Figure 9 and Figure 10.

The hand-shaped device was particularly valued by many of the participants. Despite the fact that the shape was only mimicking the shape of a human hand, they seemed to understand the resemblance: *“I noticed that it does not necessarily have to mimic a hand, for people to understand that it is resembling one. I think that is quite special”* (C14). Multiple caregivers indicated that the hand shape had an added value, because the participants interacted with it as if they were holding a human hand: *“[…] we have used heating pads before, just the cherry pit bag, but I was surprised by the fact that people were actually holding the hand. Then I thought, well the hand has added value”* (C14). The added value also became evident when looking from the perspective of a family member. She noticed the difference in the responses of her mother to different device shapes. Devices with a familiar appearance were reported to be more natural and intuitive to use and, therefore, less likely to be distracting: *“[…] with one product you could definitely notice from her response that she experienced it as more pleasant compared to the other product. With one it seemed more like she was wondering what was on her lap, but with the other one it might have felt more natural for her”* (F2).

**Care staff doubts over technological haptic devices**

*The technological devices were more likely to be associated with a “tool or instrument”. The designs and materials made them far less intuitive and uninviting to touch*.

(C4)

Almost all of the caregivers expressed their doubts on whether residents would understand the haptic sensations provided by the more technological haptic devices (e.g., the bracelet). It was explained that it was important that the residents understood the purpose of the haptic devices and the delivered haptic sensations, as it would otherwise not provide any value to them: *“You really should be able to explain what it means. My son and daughter are now sending me a touch, but they must be able to understand that”* (C10). Moreover, care professionals assumed that residents could get confused or startled by sudden vibrations: *“I think that the residents of psychogeriatric care would be a bit scared, like ‘what is happening to my body?’, I don’t think they will understand what is going to happen”* (C11). It was suggested to provide clear explanations and instruction to the residents and other users of the device to avoid such reactions: *“It requires more explanations I think, for the residents themselves and their surroundings”* (C4).

**Processing different stimuli simultaneously impedes haptic video-calling enrichment**

*“She also said: ‘I don’t know whether he is aware of the fact that he is holding something, because his focus is with the video call and that conversation alone demands quite a lot from someone’s energy’”*.

(C2)

A multitude of aspects were mentioned by care professionals and family members as requirements for creating a more personal and richer experience of video calling. These requirements included making eye contact, having a nice conversation, and sensing social connection, presence, and closeness. Care professionals and family members indicated in the interviews and focus groups that video calling, alone as well as when combined with the haptic devices, present several barriers. Residents must process the visuals, speech, and other sounds from their surroundings while interacting with the other person. According to the care professionals and family members, this can be quite a demanding experience in itself. A family member of a resident with dementia reported that sometimes the resident could end up looking restless while video calling. Thereafter, she experienced that the social connection was lost relatively quickly. 

### 4.2. Affective Level

In addition to the sensory experiences, participants from our study also reported additional affective experiences, indications of some level of calmness and relaxation and feelings of control while using the devices.

**Residents showed signs of calmness and relaxation**

*“[…]if you place that warm hand in front of them, they really do hold it and it facilitates a peace of mind”*.

(C1)

The results from the observations suggest that the haptic devices might support various residents in being more relaxed during video calls, but also if they were interacting with the haptic device without a video call. The reportedly increased levels of relaxation seem to be linked to two aspects. First, the element of sensing physical warmth appeared to be a contributor to calmness and relaxation. Participants explicitly mentioned the pleasure of sensing thermal stimuli: *“She was saying ‘I like it so much, the warmth’, and I really got the idea that it added something”* (C14). 

Second, for the participating residents from the nursing homes, the increase in calmness and relaxation when using the warm hand seemed to be caused by the form factor of the device. One of the caregivers mentioned that there had been one resident for whom holding on to hands yielded a more relaxed state of mind in general. *“He is always looking for ways to make social connection with his hands and he really likes holding hands, it offers him comfort and it makes him feel calmer”* (C3). In addition, the pressure sensation of feeling a weighted product on the skin also appeared to foster a sense of relaxation among residents from nursing homes. When the weighted collar was placed on the shoulders, the tactile stimulation seems to have a relaxing effect on the residents. A caregiver observed: *“[…] gradually it became calmer and lighter and it became comfortable for her”* and *“I witnessed the tension leaving her body.”* (C4).

**The feeling of control when using the devices**

*“I have written it down on the observation form, that people thought it was unpleasant to wear something. It is then pulled of the body, or they say things like ‘can it go away?”*.

(C14)

The abovementioned positive affective states were only observed when the haptic devices were introduced carefully and when care professionals showed the use of these devices in advance. During the try-outs, the residents from the PIMD facility, in particular, showed some hesitation in using the devices at first. After the residents had seen or used the products once, they were more likely to continue wearing or holding the devices. This indicates that how the products are introduced is an important aspect for acceptance and experiencing a positive mental state.

Residents from a care facility for older adults seemed to prefer holding the haptic devices (e.g., the warm hand or the hug robot) instead of wearing them (e.g., the weighted collar, music strap, or Hey bracelets). When discussed in the group interviews, care professionals reported that residents from nursing homes wanted to decide for themselves whether to grab something or put it back down: *“Maybe that is related to their cognitive impairments, that it is more pleasant to remain in control”* (C14). When a newly introduced device is attached to one’s arm or torso, it is more difficult to remove it when desired. Residents possibly become more dependent on care professionals to support them in using such devices. 

### 4.3. Social Level

Results from the focus groups, try-out sessions, and interviews were also analyzed in order to further understand whether the used haptic devices enhanced the social engagement between residents and family members. Below, we elucidate on the difficulties with the haptic devices and the potential focus that the use of these devices could evoke.

**Residents appear to experience difficulties in understanding the social value of haptic devices**

*“Family members weren’t sure whether there was an actual added benefit, that he is aware of its purpose, or that he just experiences the warmth and pressure as very pleasant with a calming effect”*.

(C2)

Most care professionals and family members reported that they did not clearly notice more interaction with the resident due to the haptic devices being used during the video calls: *“When the parents ask ‘what have you got there? Is it nice and warm? There is no response, so I’m unsure”* (C2). In a few situations, care professionals reported that the usage of a haptic device evoked some social effects. In their observations, caregivers described how holding a haptic device not only contributed to a pleasant feeling, but it also supported a social connection with others. For example, a care professional explained that for one of the residents, the haptic device led to social interactions with the caregiver herself, which took place during the video call: *“Every now and then she looked at me and asked if I wanted to hold it. Not because she was fed up with it, rather she really tried to connect with me”* (C14). In another situation, the haptic device served as a discussion probe during the video call: *“[…]she was a bit more communicative, she had more to talk about and was more focused on the person who sat opposite to her”* (C14). 

In particular, the Hey bracelet was mentioned to be complex to convey: *“I think it [the Hey bracelet] is valuable, but I’m afraid that it is too difficult to understand what is happening for people in nursing homes. You should really be able to explain what it entails”* (C10). Although care professionals and family members thought the concept had potential, they were hesitant in instigating the use of such devices. Interestingly, in one instance, a resident from a nursing home verbally acknowledged the haptic stimulus she received from the bracelet that was sent by her family member: *“I think it is ingenious that all this is possible. I could get that this would be real, personal contact.”* (R1).

**The use of a haptic device can support residents to focus on online conversations**

*“It really gave the resident peace of mind, a calm feeling because she held it, and she could guide her attention towards it. Residents are often distracted by what is happening around them, with the arm, she could retain focus on herself”*.

(C1)

Another way in which the haptic devices could contribute to video calling on a social level was through the possible increase in the residents’ focus on online conversations. It was reported that the music strap led to an improved focus for at least one participant with PIMD by limiting distractions from the environment. Both the parents and the professional caregiver reported from their experience that the person with PMID was better able to focus on the conversation. The music strap came with a headphone that participants had to wear. It seemed that because of the headphone, the resident was shielded from all cues other than the video call: *“[…] it resulted in an increased focus on the conversation itself, because other sounds from the environment were blocked and all that remained were sounds coming from the headphone […]”* (C1). 

Furthermore, an improvement in social engagement was noticed by a few residents. The use of pleasant handheld products during video calls was associated with a prolonged engagement in online conversations. This was especially reported for residents with dementia. Offering them a handheld product appeared to help them with literally grasping the conversation. The improvement was illustrated in two ways: through an increase of involvement in social interactions—*“I really noticed she was a bit more involved in the conversation”* (C13)—and through an increase in the duration of conversations. A family member who participated in a video call noticed: *“Normally, the attention span was about ten minutes, but now with C13 also present, we’ve talked for over one hour with the three of us and she was really actively involved in the conversation”* (F2). The overall quality of the online conversation was reportedly improved because some residents seemed less distracted, better socially engaged, and seemed supported to engage in the conversation for a longer period of time.

## 5. Discussion

The aim of this study was to explore the possibilities of various haptic devices to support residents, relatives, and care staff in intramural care facilities in times of social isolation. It was expected that the use of haptics in video calling could lead to an improved experience and emotional state for people with dementia and people with PIMD. In the present study, different product attributes of the haptic devices were evaluated by people living in three care facilities and their professional caregivers, focusing on three different levels of experiences (sensory, affective, and social). Next, we discuss our findings, strengths, and limitations and some directions for future design endeavors.

### 5.1. Summary of the Findings

Residents in long-term care facilities showed signs of calmness and relaxation during video calling, especially with the non-digital haptic devices. This resonates with earlier work showing that devices providing haptic stimulation can be effective in increasing the levels of relaxation and happiness among people with diverse cognitive abilities [18,24,54,56]. In our study, the use of a haptic device reportedly supported residents in maintaining their focus on online conversations as well as improving the interaction during video calling. Previous work has suggested that tactile stimulation can be used to improve the contact between people with dementia and their surroundings [73]. Moreover, it has been shown that for people with autism, touch therapy can lead to improvements in attention span and behavior [74,75].

Residents reported that the use of the devices improved the interaction during video calling. However, it was not clear whether residents perceived the signals provided by the haptic devices at all. When they did seem to perceive them, it was unclear if they associated the signals with their conversational partner. It should be noted that achieving this experience of social presence through a haptic device can be difficult for the larger population as well [76]. Moreover, even though residents might not have linked the signal to their conversation partner, the overall experience of social presence could still have been improved through the haptic device, as the use of this device sometimes resulted in a longer and richer conversation. Related work has suggested that the addition of a haptic signal can enhance the experience of social presence [77], supporting the promising results from our explorative study.

#### Design Recommendations

Based on our findings, we provide a set of design considerations for the design of haptic devices used during video calling for people with dementia and people with PIMD in care facilities. These design recommendations should not be seen as a one-size-fits-all solution to the design of haptic devices but rather as suggestions to consider. Residents from long-term care facilities vary in their needs, context, personal characteristics, and experiences with technology. Hence, the design requirements should be personalized and changed depending on the residents and their interest in using it.

To benefit from haptic devices in intramural care settings, these devices should have an intuitive and familiar shape and use materials that elicit tactile pleasure. These shapes contrast with haptic devices that are more technical in their appearance (e.g., the Hey bracelet, which resembles a smartwatch). Our results showed that residents favored haptic devices with form factors that were easy to hold and that appeared to convey the purpose of its use in an intuitive manner (e.g., the warm hand). In the broader field of AHDs, several design concepts have been developed utilizing human forms and organic shapes; for example, the Frebble [39], Hot Hands [78], The Hug [34], and the VibroBod [40] some of these devices have been received positively by users during user testing, others have been received critically [39,47,78]. Some of these previous designs, such as Your Gloves and MobiLimb, have been perceived as eerie because of their human-like design. It therefore seems that we should strive for a balance between intuitive and familiar designs that are not too realistic or that appear uncanny [79], such as the warm hand used in the present study. Moreover, it is important to use materials and form factors that are attractive to touch by using the appropriate materials and shapes [61]. This is in line with studies using the Crdl instrument, a wooden interactive object designed to support touch in direct personal contact between residents with PMID or dementia and their caregivers or family members [56,80,81].

Furthermore, it is suggested to move beyond vibrations as an actuation type (resonating with the manifesto of digital social touch [82]) in haptic devices in long-term care facilities. In particular, sensations such as warmth and force appear to be valued and could also increase attention toward the haptic devices. The results from our study show signs that residents were mostly interested in devices that used warmth or pressure as a sensation. This is in line with earlier findings that showed the effectiveness of weighted blankets for people with autism and dementia [56] and the use of force for affective communication [44] and haptic interactions [77,83]. Moreover, previous literature has suggested the importance of the sensation of warmth for social connections [84] as well as demonstrated positive reactions to designs that include warmth as stimulation [78].

The importance of the care context in which the haptic device is used was once more underlined during the research activities reported in this paper. Other researchers from the HCI community have discussed the challenges of designing within these contexts. Morrissey and colleagues [85], for example, explain the unique situations in care facilities, such as people cohabiting with strangers. In publicly funded long-term care units, understaffing and less involvement of family members can occur. Care professionals often lack sufficient resources, time, or tools to support residents in care facilities in engaging in meaningful activities due to the heavy workload involved in a wide range of care tasks [86]. From our study, we saw that caregivers play an important role in the access to haptic devices in intramural care facilities. Therefore, they need to have a clear understanding of and trust in the devices, and it is recommended that these be designed for ease-of-use and installation by different care personnel. In addition, to support the care staff in empowering and involving residents in enhancing the video calling, the meaning of the sensation provided by the haptic device should be as clear as possible. The use of familiar shapes and pleasant tactile fabrics can be helpful. This is in line with the concept of “Warm Technology” as proposed by IJsselsteijn, Tummers-Heemels, and Brankaert [49]. IJsselsteijn et al. [49] have proposed that technology should be familiar, non-intimating, and esthetically pleasing, as often the design of technology in the care sector is lacking in these elements. Clear and respectful instructions for the residents, the relatives, and the care staff can be of great value in this context.

### 5.2. Strengths, Limitations, and Future Research

The promising value of haptic devices for people with diverse cognitive abilities is evident. People with dementia and PIMD can face difficulties in expressing themselves through language [17] and may rely on other elements of conversations, such as touch or warmth. The lack of existing studies in this field, however, highlight the difficulties in finding the right setting and place for rigorous research practice. The current study appears to be unique in this setting and should be regarded as a practice-based exploration of an important topic. The dynamic nature of study methodologies with practice-based explorations and field research allow for a more comprehensive understanding of the practical implementation of interventions [87].The research took place when the COVID-19 measures were still in place in the participating care facilities. This factor made the research of direct value to the care sector, which was also reflected in the easy recruitment of participating facilities. However, the measures also limited the possibilities in conducting the research. Therefore, the researchers could not always be present for the data collection and had to rely on the caregivers to make notes and to fill in the observation schemes. The question that can be raised is whether the setting that was created by the care professionals was itself already positively contributing to the experience of the interpersonal contact during the video call (e.g., the professionals were present or created a specific place to call). The impact of caregivers in the design experience [88] and in the importance of guiding people, such as people with dementia, toward a new setting or “space” [85] has been reviewed in earlier work. However, the researchers provided extensive explanations to the care professionals, and through the observation schemes, they tried to ensure that caregivers were informed about which factors to focus on during the observations. A possible strength of having caregivers as observers is that they have much more experience with the participants and know more of the person, thereby having a better understanding of the behavior and its meaning compared with the researchers, who were not familiar with the participants themselves.

In general, it remains challenging to fully grasp the experiences of residents of care facilities with dementia or PIMD during a video call session. Accessing the experiences that cover the social level was particularly difficult in the current study. Additional measurements, such as physiological measurements, might be of added value to understand whether the use of, for example, headphones has secondary benefits that go beyond only supporting the focus in conversations. Could it be that presenting the voice of the other person directly into the ears of the residents creates a more intimate experience compared with solely hearing a voice coming from a screen of a tablet or laptop? It would be interesting for future research to delve into the elements that residents of care facilities need in order to create these feelings of social presence.

Furthermore, because of the complexity in collecting data during a pandemic, we were restricted in our sample size and the diversity of our sample. Lastly, it is also important to reflect on our choice to use commercially available haptic devices rather than designing the devices ourselves. The latter would have provided more room for personalizing the devices and more variety in their design characteristics for the specific user group at hand. However, it is important to note that the use of fully functional devices was deemed important despite the partial online implementation and support, and to address the urgent need for remote social contact due to the restrictive measures that were applied in care facilities during the study. Our approach was an attempt to introduce devices that have the potential to be incorporated relatively quickly within care facilities (as previously voted for by [88]). The results support the line of reasoning that solutions might not always require high-tech or state-of-the art inventions. The seemingly straightforward headphones and intuitively shaped entities were accessible and easy to use, and they played a role in shaping the experiences of residents while video calling. It has been proposed by researchers within the HCI community to strive for innovations that can also be incorporated into care facilities in a sustainable way [88]. An additional reason to consider the use of existing devices is the compliance with safety regulations of the ethics committee. The fact that the used devices are commercially available implies that these devices are in possession of a CE quality mark. Hence, we could limit the risks of failure or accidents during testing without the researchers being present. Moreover, the decision to use existing devices allowed for an exit strategy where the continuation of usage could be guaranteed even after the research ended. In the case where a haptic device was particularly liked by a resident, care facilities were able to purchase it without requiring an extensive number of resources. The ease of access is particularly valuable, given its benefits for the applicability on a large scale within the healthcare sector.

### 5.3. Conclusions

In the current study, we explored the potential of haptic devices in long-term care facilities to support people with dementia and people with profound intellectual and multiple disabilities in engaging in video calls with family members over a distance. The testing of commercially available haptic devices with different haptic stimuli showed that intuitive shapes, the use of warmth and pressure, and easy-to-use (low-tech) solutions can create additional sensory, affective, and social experiences during these video calls. Future research can further explore the possibilities of haptic devices for these contexts to enhance the experiences of residents with diverse cognitive abilities, who are an important user group for these types of technologies.

## Figures and Tables

**Figure 2:**
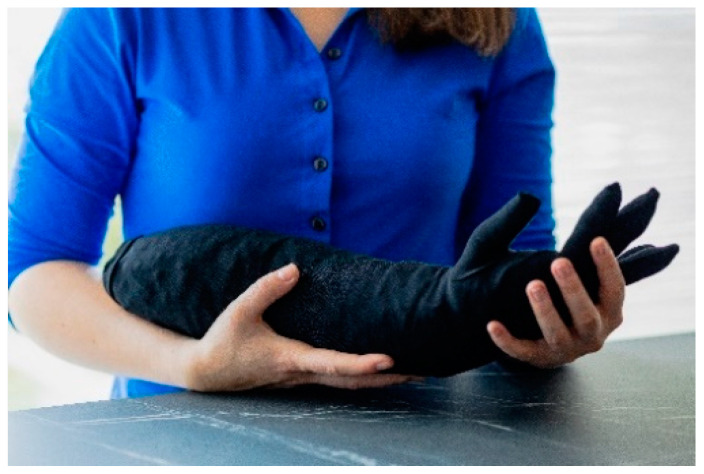
The warm hand.

**Figure 3:**
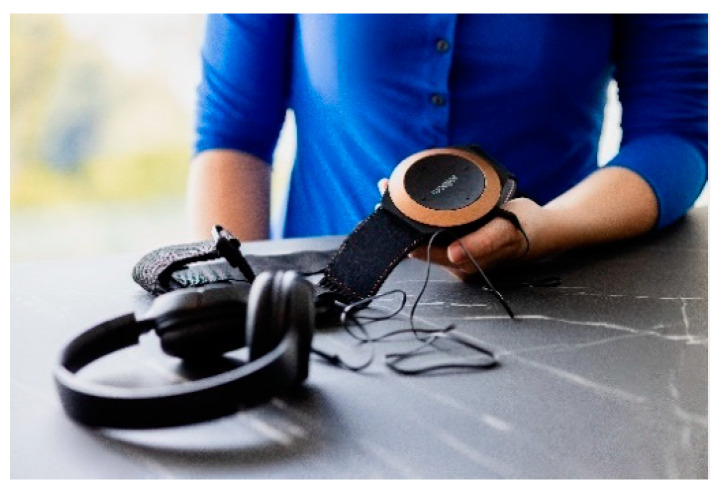
The vibrating music strap.

**Figure 4:**
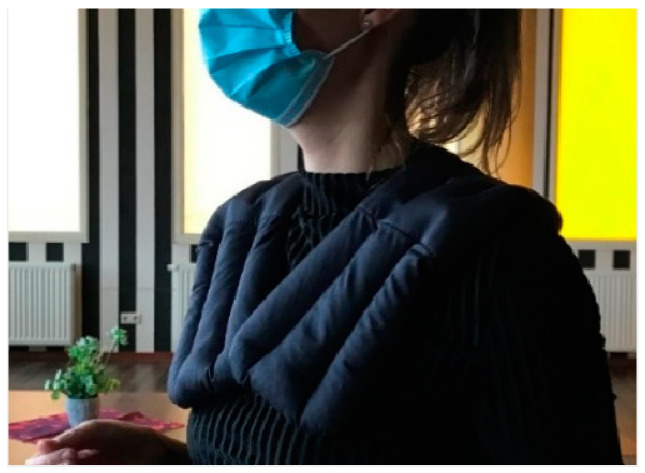
The weighted collar.

**Figure 5:**
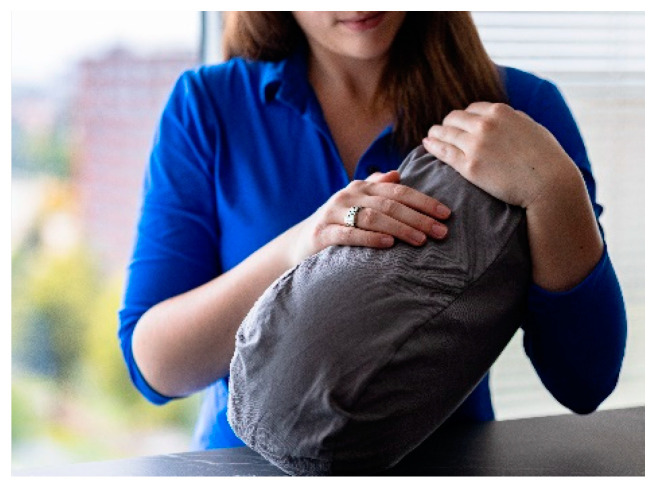
The hug robot.

**Figure 6:**
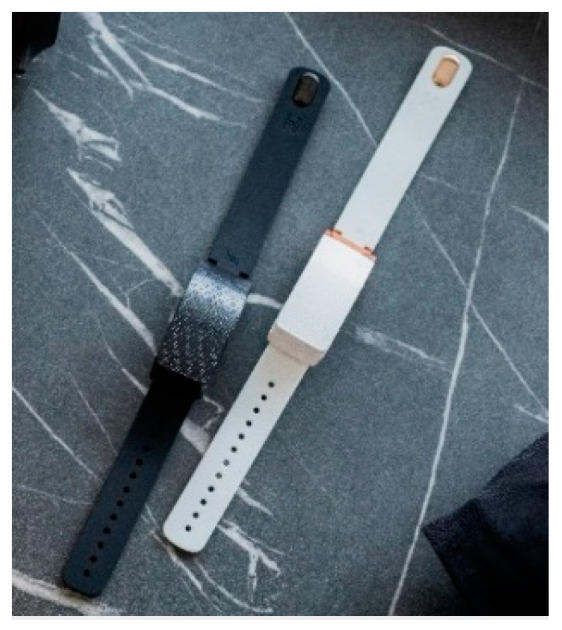
The Hey bracelets.

**Table 1:** Residents participating in the try-out sessions.

	Age	Gender	Care Facility	Prior Experience with Video Calling
R1	88	F	Nursing home	Yes
R2	82	F	Nursing home	No
R3	81	F	Nursing home	*
R4	80	F	Nursing home	No
R5	*	F	Nursing home	Yes
R6	104	F	Nursing home	*
R7	86	F	Nursing home	Yes
R8	90	F	Nursing home	No
R9	81	F	Nursing home	Yes
R10	30	F	For people with PIMD	Yes
R11	24	M	For people with PIMD	Yes
R12	23	M	For people with PIMD	Yes

* Characteristics that remained unspecified.

**Table 2:** The different stimulus materials, sorted by the type of stimuli.

Thermal stimuli	
Warm hand A pillow resembling the shape of a hand. For this study, thermal properties were added by combining a glove with a cherry pit bag. Dried cherry pits are known for their ability to retain and release heat. They are mostly used in pillows for comfort or pain relief (e.g., [64]). The shape of a hand was used as it was expected that this would be more inviting to hold. When the hand is heated, people can hold on to the hand in a similar fashion as they would with a human hand. This could stimulate the skin receptors that are sensitive to warmth, providing users with a sense of social warmth. Using thermal stimuli in the shape of a hand was inspired by the initiatives within the healthcare sector during the COVID-19 pandemic [65]. These included crocheted hands or latex gloves filled with warm water [66].	Figure 2
Vibrotactile stimuli	
Vibrating music strap The music strap by Woojer [67] is a haptic device which adds tactile cues to pre-recorded audio by using vibrotactile actuators, which could contribute to a more immersive, multisensory experience. The device has the shape of a belt that can be worn on the torso while listening to audio from different sources (e.g., games, music, or films).	Figure 3
Pressure/force stimuli	
Weighted collar The weighted collar is a fabric vest that fits around the neck and rests on the shoulders. It is made of fabric with a dense stuffing of plastic granules. When placed on the shoulders, the collar applies deep pressure to the back, shoulders, and chest, which are areas of the body that are also targeted during interpersonal touches, such as when hugging or during touch therapy. The use of a weighted vest was inspired by similar items that are already known and used within healthcare settings. The weighted products currently in use, such as vests or blankets, are associated with positive effects on the mental states of people living with autism, anxiety, or dementia [56].	Figure 4
Hug robot The Somnox [68], used as hug robot, was originally developed as a sleeping aid. The Somnox mimics the breathing rhythm of a human. Users can feel this rhythm through its movement. Feeling this simulated breathing pattern can result in relaxation. Therefore, it could provide an interesting addition to pet robots.	Figure 5
Hey bracelets The Hey bracelet [69] is an AHD that was especially designed for couples in a long-distance relationship. However, the Hey bracelet might also be valuable for other use cases where interpersonal touch is scarce. If a person touches the bracelet, it sends a touch signal to the connected bracelet. The person wearing the connected bracelet will then feel a squeeze, followed by a vibration (the straps of the bracelet will become tighter).	Figure 6

## Data Availability

Data are only available on request due to privacy restrictions. The data presented in this study are available on request from the corresponding author. The data are not publicly available due to the sensitivity of the data and privacy regulations.

## Public Involvement Statement

Care professionals, residents of long-term care facilities and family members were involved in the study in order to gain first-hand insight into the potential of haptic devices in long-term care. Eleven care professionals participated in focus group sessions and six care professionals participated in semi-structured interviews after they conducted the observations of residents during Try-out sessions. In these Try-out sessions, twelve residents of long-term care facilities participated. Two family members were willing to share their experiences and views of the try-out sessions in a semi-structured interview.

## Guidelines and Standards Statement

This manuscript was drafted according to the guidelines of qualitative research.

## Appendix A

Interview guide for sessions with care professionals, translated into English.

### Introduction

Selection Procedure—Gaining insight into how the care staff selected the participants. How was the choice made, and when was someone deemed suitable or not? There is evidence of sample bias, but how does this arise, and what are the possible consequences for the results?

- Can you tell me about how you selected the residents for the study? Based on what criteria, for example?
- Can you estimate how residents who were not selected might have reacted?
- Were there selected residents who declined to participate?
- What kind of responses might you have received if these residents had participated?

Materials—Understanding which products were tested by the care professionals, residents, and their relatives. Were the products used once or multiple times, and why was this chosen? How was the selection of products and their use determined?

- Which products did you try out with residents?
- Can you explain why you specifically tried these?
- Why not the others?
- How often was this product tested with each resident?

Context—A description of the context of the trials. For instance, was it a quiet room where the products were tested, or was it a large space where other residents were also present?

- In which setting did the trials take place? Was this different for each resident?
- Who was present at the time of the trials?
- What activity took place before the trials?
- How long did a trial session last?
- Were multiple products tested in one session?
- How were the products tested? Handed over or placed on the table, for example? Can you elaborate on this?

General Evaluation—Understanding how the past few months have gone; what went well during testing, and what did not? Did the care staff encounter any issues?

- How did the testing go in the past weeks?
- What role did the products play in interacting with others? In communication?
- Were the products of value for residents’ interaction in general? Can you explain how?

Observations—Inquiring about observing residents during the trials. Did they manage to document everything according to the observation scheme? What did they observe specifically? And what does that tell us about the products on the three different levels (sensory, affective, social)?

- How did the observations go?
- What were the initial reactions observed from residents regarding the products? (per product)
- Did the reactions change during the trials? (per product)
- Did you notice any reactions, verbal or non-verbal, that stood out?
- What kind of reactions were these, and what made them stand out?
- How did residents react to touching the products? (per product)
- What feelings or emotions did the residents display during the trials? (per product)
- Are the products approached as a means of social contact? (per product)
- Can you explain how you noticed this?

Reflection—Looking back on the trials, what is the final assessment of the products? Can the products contribute to warm contact for residents according to the care staff, or is something else, or more, needed? Has the care professionals’ perception of the products changed after the trials?

- Could the products contribute to the feeling of connection with a family member or was this not the case?
- Which product did or did not contribute?
- In what way did the product contribute?
- Can you explain how you noticed this?
- Did the residents react to something that happened during the video call afterward?
- Was the reaction different from a “normal” video call?
- If yes, can you explain what was different?
- Did the residents feel more connected to the family member during the call, or not at all?
- How did you notice this?
- Have you personally tried the products?
- What is your opinion on the effectiveness of the different products after trying them?
- Do you have any ideas about what could be tested or tried in the future?
- Can we approach the relatives of residents for a brief interview about their experiences?
